# Surface Connectivity and Interocean Exchanges From Drifter‐Based Transition Matrices

**DOI:** 10.1002/2017JC013363

**Published:** 2018-01-19

**Authors:** Ronan McAdam, Erik van Sebille

**Affiliations:** ^1^ Grantham Institute‐Climate Change and the Environment Imperial College London London UK; ^2^ Department of Physics Imperial College London London UK; ^3^ Institute for Marine and Atmospheric Research Utrecht Utrecht University Utrecht The Netherlands

**Keywords:** transition matrix, interocean exchange, surface transport, transport barriers

## Abstract

Global surface transport in the ocean can be represented by using the observed trajectories of drifters to calculate probability distribution functions. The oceanographic applications of the Markov Chain approach to modeling include tracking of floating debris and water masses, globally and on yearly‐to‐centennial time scales. Here we analyze the error inherent with mapping trajectories onto a grid and the consequences for ocean transport modeling and detection of accumulation structures. A sensitivity analysis of Markov Chain parameters is performed in an idealized Stommel gyre and western boundary current as well as with observed ocean drifters, complementing previous studies on widespread floating debris accumulation. Focusing on two key areas of interocean exchange—the Agulhas system and the North Atlantic intergyre transport barrier—we assess the capacity of the Markov Chain methodology to detect surface connectivity and dynamic transport barriers. Finally, we extend the methodology's functionality to separate the geostrophic and nongeostrophic contributions to interocean exchange in these key regions.

## Key Points

- The error within probabilistic ocean surface transport models is highlighted
- Fluxes at key interocean exchanges are recreated within the model
- The role of geostrophic flow in interocean connectivity is explored

## 1 Introduction

Knowledge of the connectivity between different parts of the ocean is crucial for predicting the location of missing objects, pollution, and biogeochemical tracers. Floating litter is one such oceanographic tracer which is abundant and harmful throughout the global marine environment (Barnes et al., 2009; Cózar et al., 2014; Thompson & Gall, 2014). Due to the practical difficulties involved, in situ ocean observations of debris are sparse. This is particularly true for litter and is an obstacle for determining the risk to ecosystems and humans (Hardesty & Wilcox, 2017; Law et al., 2014). To track the pathways taken by floating debris, fill in gaps in the spatial record, and identify regions of relatively high concentrations, ocean surface transport models are used (Hardesty et al., 2017). For example, Lebreton et al. (2012) used a hydrodynamic ocean surface model seeded with virtual floating particles, and forced by observed atmospheric parameters such as wind speed.

An alternative method has been developed that is based on observations of floating drifters. Maximenko et al. (2012) and Van Sebille et al. (2012) both use a probabilistic approach to determine tracer pathways. In these methods, the global flow field, derived from a global data set of satellite‐tracked drifters, is represented by a probability distribution function describing quantities of tracer transport between one area to another. A study comparing the Lebreton et al. (2012) hydrodynamic model with the probabilistic models found that each model's resulting basin‐scale tracer accumulations were similar (Van Sebille et al., 2015). In other words, they adequately represent global and decade‐scale transport phenomenon.

This probabilistic approach requires the partitioning of the ocean into segments, or cells. In previous studies, each segment has been a square cell of resolution *dx*, with *dx* typically 1°–2°. Using a data set of trajectories, the number of connections between cells can be determined—for a time step *dt*. Satellite‐tracked drifters provide the trajectory data, ensuring all phenomena acting on floating objects are incorporated into the model without the need for parameterization (e.g., a stochastic term to represent eddy diffusivity; Berloff & McWilliams, 2003). A Markov Chain simulation is initiated by “releasing” tracer into a cell, or group of cells. The output of the model can be interpreted as a probability map for a single particle, or as the dispersion of a cloud of tracer.

This approach is also known as the transfer operator method, the transition matrix method or a Markov Chain model. It has widespread benefits, particularly of reduced computational speed and reduced data storage compared to primitive‐equation circulation models (Khatiwala et al., 2005) and can even be hosted on a website for widespread accessibility and rapid repetition of experiments (Van Sebille, 2014). The approach is used to decide when and where drifter coverage targets are not met, and to devise deployment strategies (Lumpkin et al., 2016). Subsurface Argo float pathways have also been analyzed with transition matrices (Sevellec et al., 2017).

**Figure 1:**
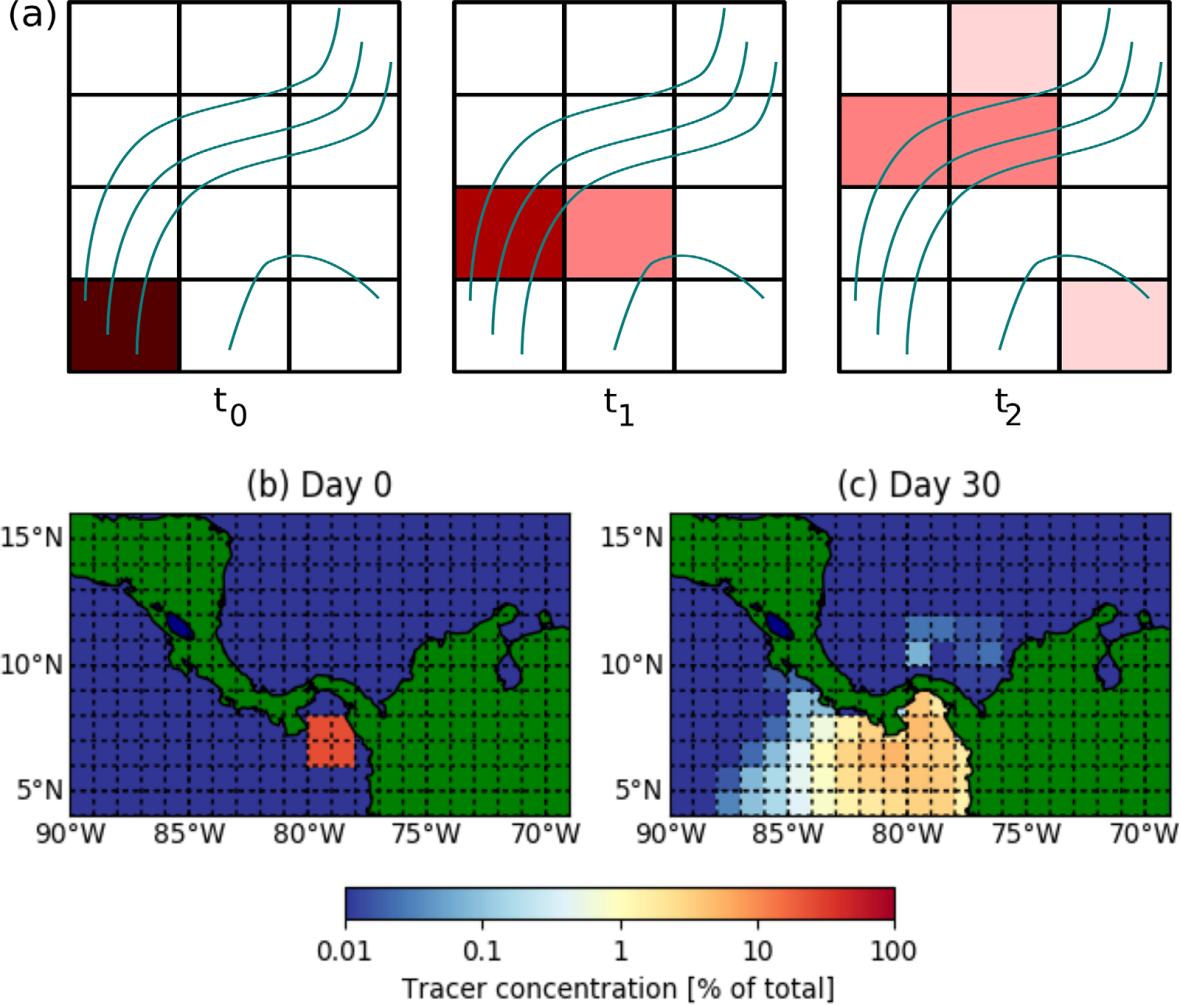
Artificial dispersion in a Markov Chain ocean model. (a) Schematic highlighting how spurious connections in cells can connect distinct flow regimes (blue trajectories). (b) Tracer transport model of flow around a geographic transport barrier (Panama Isthmus). Tracer is released around (7°N, 79°W), and despite the physical barrier between oceans, crosses from the tropical Pacific Ocean into the Caribbean Sea. As the land barrier is narrower than a grid cell, both waters coexist in cells near the land. After 30 days, 0.3% of tracer has leaked.

Despite the benefits and potential for Markov Chain model applications, there are problems inherent to this representation. The results of Markov Chain models are sensitive to the details of the gridding: a trajectory which briefly passes into a corner of a cell will lead to the Markov Model filling the entire area represented by the cell with some tracer. One cell may contain different flow patterns, which are physically unconnected, yet, due to the finite cell size, are artificially connected in the model (Figure 1a). Hence, there can be dispersion between regions which is artificial, and this artificial dispersion has been found to depend on the grid size used (Rypina et al., 2016). An extreme example of artificial dispersion occurs near narrow land masses (Figure 1b). Due to the lack of inbuilt memory in the model, some tracer can “forget” its origin in the Pacific Ocean and “leak” across Panama into the Caribbean (and vice versa).

While the above case of the Panama Isthmus is an extreme example used for illustrative purposes, it raises concern that oceanographic transport barriers, which mark the boundary between two poorly connected flow regimes, may display artificial connections in the Markov model. Most of the ocean on scales larger than the mesoscale is in geostrophic balance. Boundary currents and their extensions, where the geostrophic balance also dominates, are very often transport barriers. There is indeed ample evidence of strong currents acting as barriers to surface drifters. The Gulf Stream and North Atlantic Currents block surface transport between subtropical and subpolar gyres (one drifter has traversed this barrier in 30 years of observational records; Brambilla & Talley, 2006; Hakkinen & Rhines, 2009). Poleward surface transport across the North Atlantic Current occurs either in a narrow near‐shore flow (Rypina et al., 2011) or at depth (Burkholder & Lozier, 2011). The Antarctic Circumpolar Current and Kuroshio current have also been studied with drifter data, highlighting the role played by fronts in restricting drifter movement (Niiler et al., 2003; Trani et al., 2014).

Submesoscale eddies and other ageostrophic processes (including wind‐slip and Ekman transport) may act as conduits across geostrophic fronts. Moreover, geostrophic eddies, which are formed within boundary currents and later split away, can trap drifters and carry them away from the geostrophic front. However, it is not yet clear how much of this cross‐frontal transport is driven by geostrophic mesoscale eddies and how much by nongeostrophic processes. Determining the influence of velocity field components is crucial for understanding the connectivity between parts of the ocean; Maximenko et al. (2009), for example, identified the role played by nongeostrophic velocities in gyre‐scale accumulation.

In this study, the ability of Markov Chain models to recreate interocean & cross‐boundary transport is assessed. Moreover, the applications of this method are extended to determine the contribution of geostrophic velocity field components to the transport pathways, and to build maps of connectivity. In section 2, we combine global drifter data sets with measurements of absolute dynamic topography (an indicator of the geostrophic flow component) while describing the matrix construction. In section 3, a sensitivity analysis is performed on the transition matrix parameters—in an idealized test‐case and for the real ocean. The test‐case serves to improve understanding of the effect of artificial dispersion on prediction quality. In section 4, we apply the transition matrix method to two case studies of interocean transport; the Agulhas Current System and the North Atlantic subtropical and subpolar gyres. Finally, the results are discussed and conclusions are presented in section 5.

## 2 Data Sets and Method

### 2.1 Global Drifter Data Set: Current State

The NOAA Global Drifter Program (GDP) drifters cover the majority of the global surface ocean, albeit unevenly, with data available from 1979. In section 2.2, we interpolate satellite‐altimetry products to drifter coordinate positions, therefore we use drifter data from 1993 and onward for the analysis here. Coordinate positions are taken several times per day, depending on satellite positions. They are then interpolated to provide a 6‐hourly data set. An augmented version of the data set is now available with hourly interpolated readings (Elipot et al., 2016) but here we use the original 6‐hourly data as these are sufficient temporal resolution for our goals (see section 2.2).

Coverage is a function of drifter deployment and the convergent nature of two‐dimensional surface flow. Within the data set, there are two different types of drifters—drogued and undrogued—whose current‐following abilities, and therefore their destinations, differ (Beron‐Vera et al., 2016). Undrogued drifters, which make up 50% of the total, have lost their 15 m depth water‐following drogue and are more susceptible to wind‐slip (Niiler et al., 1995). Here we use all available drifters to allow for maximum coverage, acknowledging that this represents a 15 m depth‐averaged flow field.

**Figure 2:**
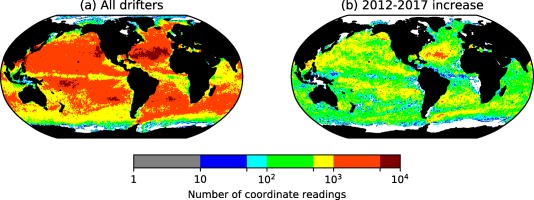
Drifter data coverage. (a) Number of historical GDP drifter coordinate readings per 1° cells measured from 1979 to March 2017. Areas in white contain no coordinate readings. (b) The increase in the number of readings since 2012 (the difference between March 2017 values and 2012 values). Areas in white have not seen an increase in readings.

Coordinate positions are accurate within 15 m to 1 km, depending on the constellation of the satellites hosting the Argos or the Global Positioning System (Lumpkin & Pazos, 2007). For studying transport between ∼1° resolution grid cells, this is negligible. However, the quality of a transition matrix representation depends heavily on the availability of trajectory data. Key areas of interocean exchange—such as the Indonesian Throughflow, the Arctic Ocean and much of the Antarctic Circumpolar Current—contain orders of magnitude fewer drifter readings than the center of ocean basins (Figure 2a).

Since the first global transition matrix study (Maximenko et al., 2012), the number of available historical drifters has doubled to roughly 20,000. Since the creation of the plasticadrift.org online tool (Van Sebille, 2014), the number of coordinate readings has increased from 24 million to 34 million. Areas where this increase has occurred mostly include previously well‐covered regions (Figure 2b); this is due to the domination of ocean current dynamics over deployment in determining coverage. Lumpkin et al. (2016) used transition matrices to determine the most effective deployment areas to fill the gaps of low coverage yet it is too soon to see the benefits of this new methodology.

We highlight two regions with distinct roles in the global ocean circulation, for use in later case studies, which contain a relatively large amount of data: the Agulhas system and the North Atlantic.

The Agulhas region, south of Africa, is flanked by regions of high drifter coverage. The Agulhas Current, which begins between the continent and Madagascar, is a rapid current which follows the coast. At the southernmost tip of the continent, the current reaches its retroflection point: here the Agulhas Current splits into an eastward Agulhas Return Current and the north‐westward Agulhas leakage. Due to the high velocities in this area, drifters spend a short amount of time within the current, reducing the coverage (Figure 2a). Despite the intense divergent dynamics, recent drifter coverage has remained similar to more convergent parts of the ocean, implying drifters are consistently entering the Agulhas leakage (Figure 2b).

Within the North Atlantic, the Gulf Stream acts as a surface transport front between the subtropical and subpolar gyres (Brambilla & Talley, 2006; Rypina et al., 2011). Despite the presence of a surface transport barrier, there is a very large poleward heat transport, confirmed by both theoretical arguments (e.g., Marshall et al., 2001) and observations (Johns et al., 2011); much of this transport is believed to occur in the subsurface along isopycnals (Burkholder & Lozier, 2011). The surface flow on both sides of the Gulf Stream, as well as downstream, has been relatively well sampled (Figure 2a). The exception, in this region, is the Labrador Current, where low coverage may be a result of a historical lack of deployment in the area between Canada and Greenland. There have been recent attempts to increase deployments in this area (supporting information Figure S1).

### 2.2 Transition Matrices

The theoretical treatment of constructing transition matrices has been thoroughly covered within oceanographic studies (e.g., Ser‐Giacomi et al., 2015). Here we provide a brief descriptive overview.

The domain, in this case the entire Earth including land and ocean, is gridded into a rectangular network of cells (with a cell size *dx* × *dx*). Then, each drifter trajectory is split into subtrajectories of length *dt*. For each subtrajectory, the initial cell *i* and final cell *j* are determined. The number of subtrajectories which begin in a cell *i* and end in a cell *j* as a ratio of the total to leave cell *i*, are normalized to a row‐sum of 1 and thus return a probability. The probability distribution function (PDF) for cell *i* therefore describes the probability of going to any other cells in a time *dt*. This is used as a proxy for the proportion of tracer transferred between the cells.

PDFs are tabulated into a transition matrix *T_dt_*. Meanwhile, the quantities of tracer in all cells are stored in the elements of a row vector *R*. The evolution of *R* into a future state is achieved by the vector‐matrix multiplication:

(1) $$R^{t+dt}=R^{t_{0}}T_{dt}$$

The key parameters—trajectory length/time step (*dt*) and cell size (*dx*)—have not been used consistently between, nor compared within, oceanographic studies. An exception is Froyland et al. (2012), who compared the size of Agulhas rings in their regional transition matrix model based on virtual particles, using two different time steps. For the purposes of detecting local coherent structures, the difference was negligible.

We use typical cell sizes from the literature (0.5° and 1°) as well as a coarse 2° cell size to properly highlight the sensitivity. Using cell sizes smaller than 0.5° not only slows down the calculation but also increases the number of cells with no coverage. A proportion of drifters may remain within a cell after a time step *dt*, leading to nonzero values in the transition matrix diagonal.

In Markov Chain theory, evolving a system with a transition matrix (*T_dt_*) to a given time while using different time steps should return the same output. For example, four multiplications of $T_{dt=5\;\text{days}}$ should be equivalent to one evolution with $T_{dt=20\;\text{days}}$. Generally, the following statement should be true:

(2) $$T_{dt}^{n}=T_{ndt}$$

The dispersion shown in Figure 1 caused by spurious connections, depends on the subtrajectory coordinate readings used to build the transition matrices. Using different time steps will provide different subtrajectories and so the nature of the artificial dispersion will be different. Hence, the model parameters used may provide different results and may control the accuracy of the model. Given that different time steps might be used for the same application (i.e., tracking plastic debris pathways on a weekly basis as well as seasonally), testing (equation (2)) is an important task. In the following sections, we will compare transition matrices for the following time steps, expanding on the range used in the literature: *dt* = 5, 20, 60, and 180 days. In previous studies, the choice of *dt* has mostly been a matter of practical preference of whether to visualize tracer transport at weekly or seasonal outputs.

It is important to make clear that the concepts of grid resolution and model time step do not correspond to those of primitive‐equation solving models. For example, regardless of the cell size used, the trajectories used to construct *T_dt_* have sampled the full‐flow field, including submesoscale turbulence. A primitive‐equation model would need a sufficiently fine resolution to represent this motion.

### 2.3 Geostrophic Drifter Subsets

The surface flow field, as experienced by drifters, is driven by geostrophic currents and eddies, as well as wind‐slip, Ekman transport, Stokes drift, and submesoscale processes. One way to study transport across fronts due to nongeostrophic processes is to compare the geostrophic and full velocity fields. In existing methodologies, Poulain et al. (2012) applied a low‐pass filter to the velocity frequency spectrum while Maximenko et al. (2009) derived the geostrophic velocity field from sea surface height. However, neither study calculated the tracer transport due to these velocity fields. Here we aim to compute these geostrophic transports directly from drifters.

Here we extract a subset of observed drifters whose trajectories are dominated by geostrophic flow, by computing their tendency to flow along contours of absolute dynamic topography (ADT). We map each drifter coordinate reading to an ADT value, using daily ADT maps from 1993 onward. Due to the overlap in satellite orbits, the effective resolution for the sea level anomaly is 2.0° (Fu et al., 2010). However, the AVISO products are provided at an interpolated resolution of 0.25°. To estimate the ADT reading at each drifter coordinate, we used a linear, 2‐D interpolation of ADT values to the drifter coordinates. The error in ADT readings is the combination of the error in the geoid and mean sea level anomaly (MLSA). The geoid is modeled at 100 km resolution to an accuracy of 1 cm (Mulet et al., 2012). The mapping error in MSLA is between 10 and 20 cm within the boundary current systems (i.e., Agulhas and North Atlantic) but less than 5 cm throughout the majority of the ocean surface (MSLA error maps available from AVISO MSLA products via Copernicus Marine Environment Monitoring Service; supporting information Figure S2).

**Figure 3:**
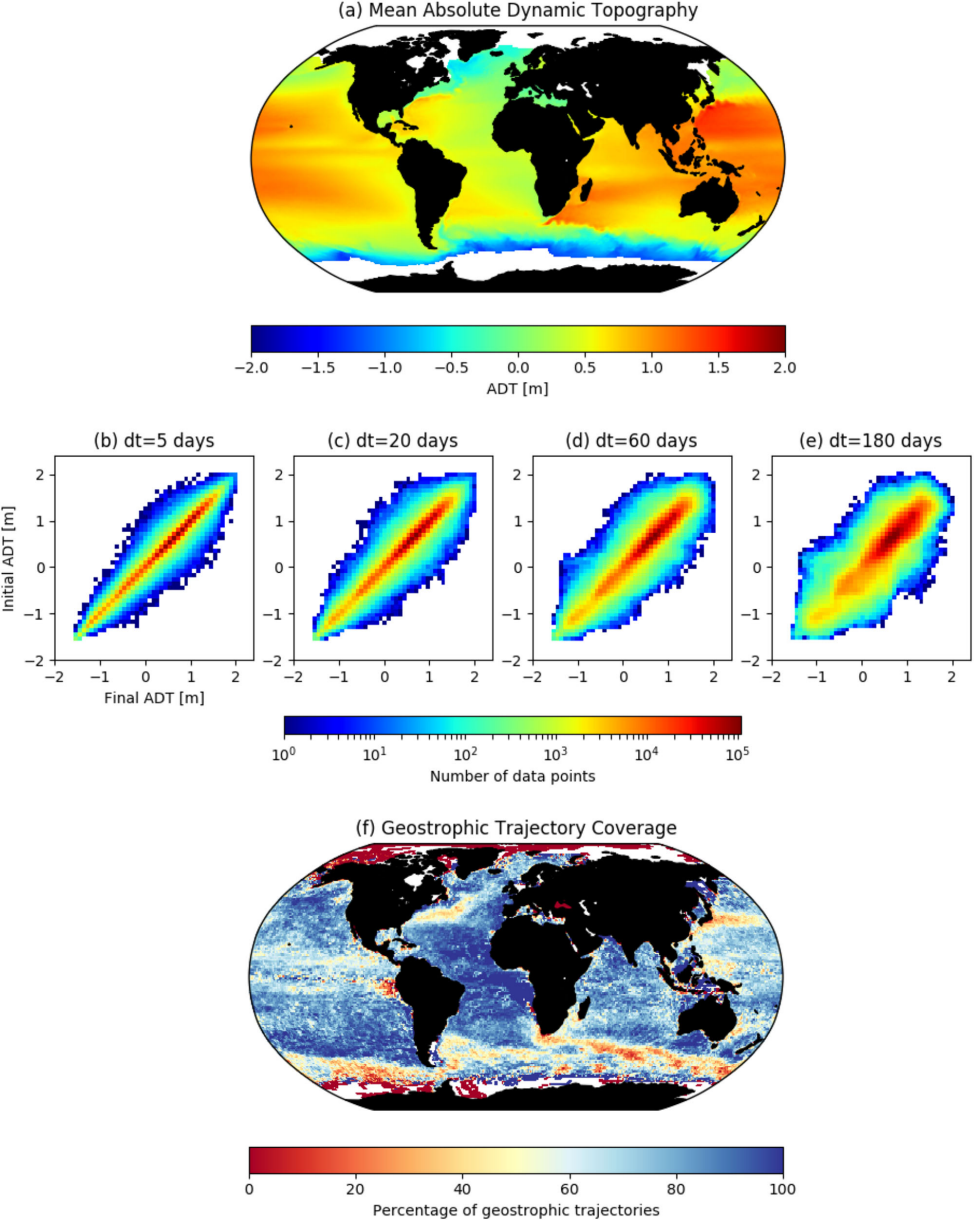
Mean absolute dynamic topography (ADT) experienced by surface drifters. (a) 30 year average of ADT on a 0.25° grid, extracted from the interpolated AVISO data set. (b–e) Change in ADT at beginning and end of all drifters subtrajectories of length *dt*. The sparseness of the connectivity matrices indicates the tendency of drifters to change ADT values. (f) Map of the proportion of subtrajectories whose ADT values stay within 0.2 m over a 60 day time scale.

The range of ADT values experienced by drifters contains information about the extent to which drifters follow geostrophic contours. For each of the subtrajectories in the drifter data set ( $T_{5},T_{20},T_{60},T_{180}$), we compare the initial and final ADT values. The before‐and‐after comparison is displayed by means of a connectivity matrix (not to be confused with the transition matrices; Figures 3b–3d). In this analysis, an identity matrix would describe drifters which remain on the same ADT contour—indicating purely geostrophic flow. Deviation from the diagonal matrix represents a reduced likelihood of approximately following geostrophic contours, for the given time step *dt*. For all time steps, there is a majority of datapoints along the diagonals: the number of subtrajectories which remain with 0.2 m of their initial ADT value are 98.2%, 94.7%, 87.9%, 72.4% for 5, 20, 60, 180 days time step, respectively. As the time step increases, the sparsity of the matrix is reduced; given a longer drift time, drifters will finish in a wider range of ADT values. Below seasonal scales (Figures 3a and 3b), the matrices are less diagonal in the upper right corner of the plot: apparently, drifters in positive ADT regimes are more likely to experience a sharp change in ADT value. The regions where drifters are more likely to experience greater ADT change are in the boundary current (Figure 3f). Reasons for this may be that these are the regions with the highest ADT values and sharpest gradients but also contain mapping error in these regions (Figures 3a and 3f and supporting information Figure S2). Though the ADT change is dependent on time scale, these regions remain coherent regardless of time scale (supporting information Figure S3).

Drifter trajectories do not strictly follow ADT contours, due to the presence of nongeostrophic contributions. Hence, to construct geostrophic transition matrices, we select subtrajectories which remain within a narrow range of ADT values. The definition of geostrophy‐following drifters in this method is therefore a function of the chosen range. The error in MSLA measurements is below 20 cm across the global ocean, so this acts as a minimum threshold to our range. To ensure the selected trajectories are predominantly driven by geostrophy, it is necessary to use the narrowest range possible.

A trendline is calculated for the *dt* readings between cells *i* and *j* (section 2.2). Our definition of a geostrophic trajectory is therefore one with a gradient (*m*) which obeys the following inequality:

(3) $$m<\frac{dADT}{dt}$$

Using a trendline smooths the error signal and ensures the definition of geostrophy is independent of time step. Subtrajectories may still be misidentified if erroneous ADT values dominate the signal on the scale of the time step. Thus, misidentification may be more likely for the shortest time step (i.e., $T_{dt=5days}$). It is hypothesized that misidentification of geostrophic subtrajectories as nongeostrophic, and vice versa, will approximate a random sampling of the total data set of subtrajectories.

**Figure 4:**
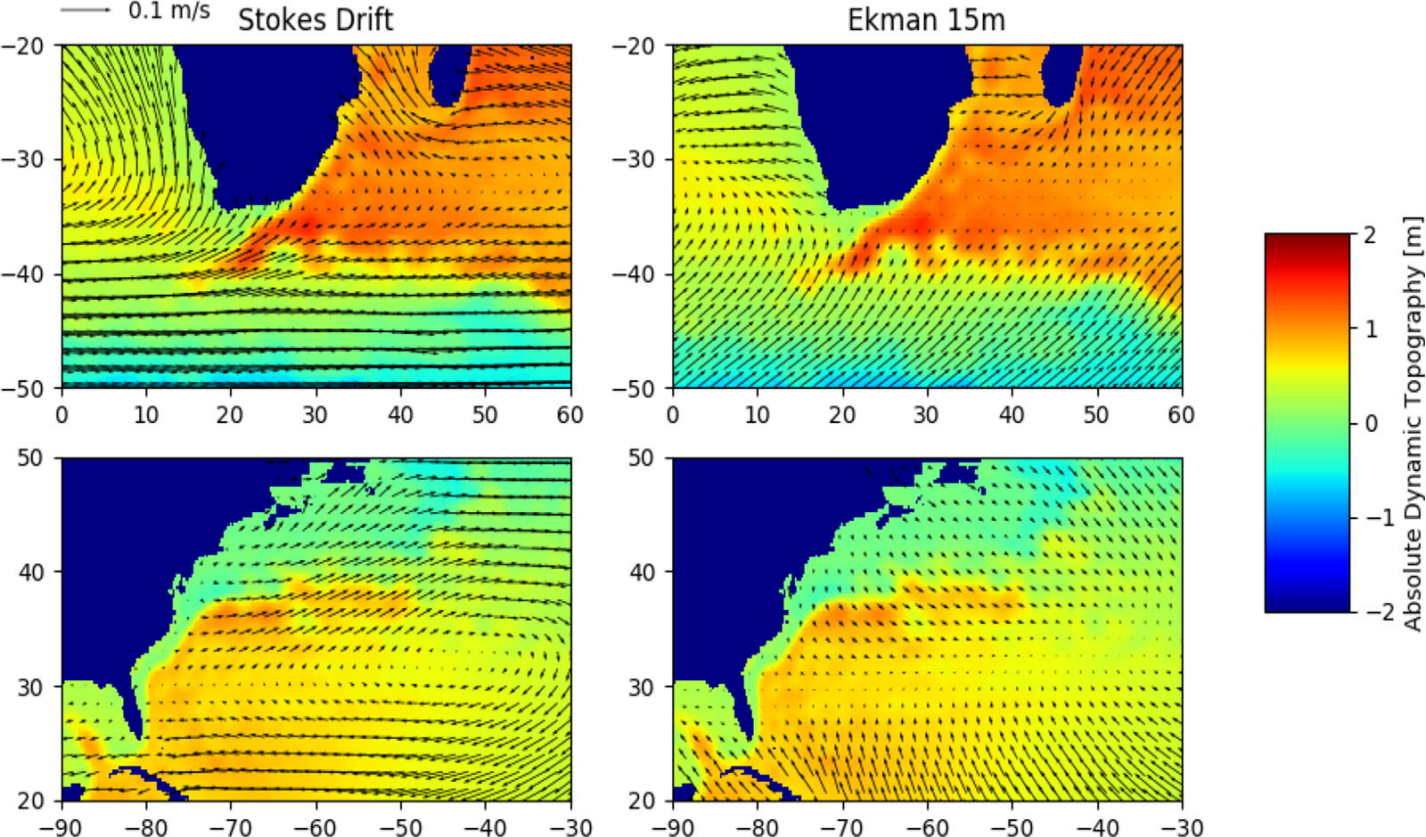
Alignment of nongeostrophic and geostrophic velocity fields. (left) Stokes drift and (right) 15 m Ekman velocity vectors are plotted over AVISO absolute dynamic topography. The Stokes drift 0.5° field is extracted from European Centre for Medium‐Range Weather Forecasting (ECMWF). The Ekman transport 0.25° field is extracted from the GlobCurrent data set. Arrows are plotted every 1.5° for clarity. All fields are annual averages from 2014.

Misidentification may also occur if nongeostrophic phenomena induce transport along contours of ADT. Stokes drift (data extracted from the WaveWatch3 hindcast with ECMWF winds; Dee et al., 2011; Tolman et al., 2002) and Ekman transport (data produced by the GlobCurrent project; Rio et al., 2014) are two such phenomena; here they are compared to the ADT field average, from 2014, in the Agulhas and North Atlantic basin regions (Figure 4). In the Agulhas Current, Stokes drift occurs along ADT contours but at angles between 90° and 180° to the current direction (i.e., opposing the geostrophic flow) while the Ekman transport is mostly negligible. In the Return Current, Stokes drift is also eastward but the average field does not meander sharply like the ADT contours. In the southeast corner of the South Atlantic gyre, Ekman flow occurs in the same direction as the advection of Agulhas rings (northeastward) but these structures have already been formed at around (20°E, 40°S; Figure 4, top). In the North Atlantic gyre, the Ekman field is largely perpendicular to ADT contours (Figure 4, bottom). Crucially, along the Gulf Stream, Stokes flow is directed westward but at the North Atlantic Current, much like in the Agulhas Return Current, it does not experience the sharp meandering of the ADT field.

Lastly, undrogued drifters are more susceptible to wind‐slip (Beron‐Vera et al., 2016) and also experience the surface Ekman field (as opposed to the 15 m depth field). As a result, it may be expected that undrogued drifters are not in geostrophic balance, and fewer of this type will obey Equation (3). However, while the proportion of which obey equation (3) is less than the corresponding proportion of drogued drifters, the difference is always less than 2% for all time steps (supporting information Figure S4 and supporting information Table S1). Thus, undrouged drifter motion appears to also be dominated by the geostrophic balance and allows the use of the full GDP data set in constructing geostrophic transition matrices.

## 3 Sensitivity Analysis

### 3.1 Idealized Subtropical Gyre System

Before applying the model to observed drifters, we quantify the effects that transition matrix parameters have on the model output by producing a Markovian representation of a purely advective flow with an analytical solution. No stochastic dispersion term is added to represent subscale ocean dynamics (e.g., Berloff & McWilliams, 2003), ensuring that any deviation in tracer pathways from the analytical flow will be inherently artificial.

We choose the western‐intensification subtropical gyre system of Stommel (1948), as the quality of the numerical solver used (fourth‐order Runge‐Kutta) has been tested with this scenario (Fabbroni, 2009). The stream function is defined as

(4) $$\psi =A\sin(\frac{\pi y}{b})\left[\exp^{(x-b)\pi /b}+\exp^{xb/\pi }-1\right]$$

where *A* is a constant which incorporates the model wind stress, friction, and domain size. The domain size is b=10,000 km in both the zonal and meridional directions. Using $A=10^{6}$ m yields velocities which peak at ∼ 50 cm/s in the western boundary current and ∼ 5 cm/s in the eastern gyre interior.

**Figure 5:**
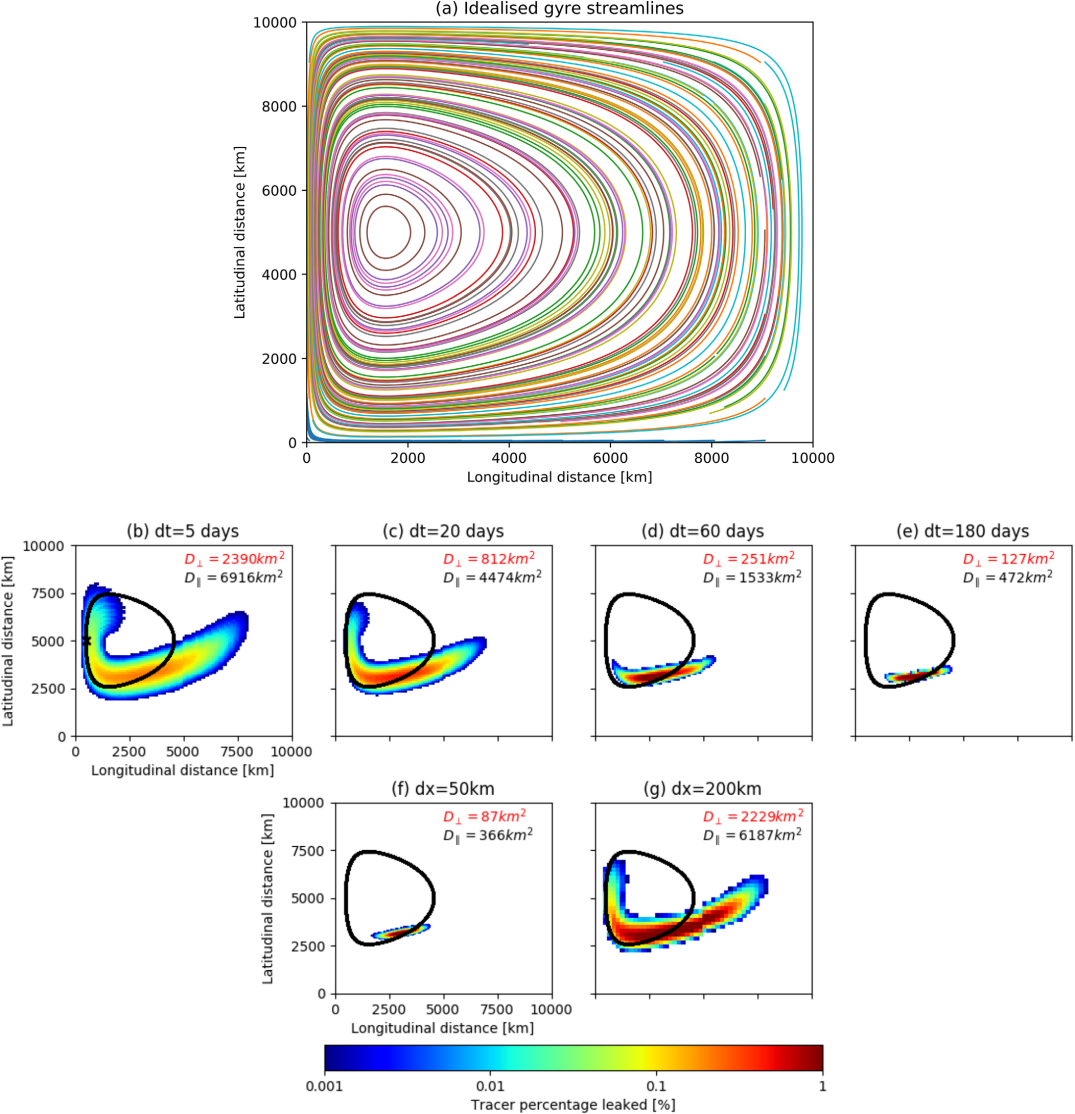
Stommel western‐intensification flow: applying the Markov Chain model to an analytical case study. (a) Trajectories of virtual particles, used to construct the transition matrix, are initialized at 100 km spacing in both latitudinal and longitudinal directions and integrated for 10 years. (10% of the streamlines particle trajectories are shown for visual purposes). (b–e) Model output comparison for a range of time steps (with constant cell size dx=100 km). (f–g) Model output comparison for a range of cell sizes (with constant time step dt=60 days). Tracer is released at (500, 5,000 km; black cross (Figure 5b)). The streamline corresponding to the tracer release cell center is shown in black, to illustrate the difference from the analytical solution and is used to compute the dispersion components. The perpendicular and parallel components of dispersion (equations (5) and (6)) are shown in red and black text, respectively.

Trajectories are calculated with the OceanParcels Lagrangian analysis toolkit (Lange & van Sebille, 2017). The grid is seeded throughout a 2‐D horizontal plane, with particles initialized at time t=0 s every 100 km (providing 10,000 particles). The velocity field is integrated with an RK4 time step of 6 h and saved every 24 h; particles are advected for 5 years (Figure 5a). Although this is not enough time for the outermost particles to complete a full circuit, the seeding is sufficiently dense and the integration time sufficiently long so that all cells are connected to at least one other cell.

The transition matrices are created in the same way as for the ocean‐based drifters (section 2.2). In this case study, however, we define spatial resolution in kilometers, using 50, 100, and 200 km. These approximately correspond to the sizes of cells used in ocean Markov models (0.5°, 1.0°, and 2.0°). The time steps used are the same as in section 2.1. Tracer is released within the western intensification and tracked over a 7 year period, which captures the journey from the boundary current into the slower, eastern section of the domain—in anticipation of our study of the North Atlantic.

First, we study the effect of *dt* (Figures 5b–5e) on the model output (keeping *dx* constant). At every multiplication, some leakage into adjacent cells occurs. Evolving the system to a given time with a smaller *dt* will require more vector‐matrix multiplications. Therefore, error accumulates more rapidly in models with smaller *dt*. The artificial dispersion causes the tracer cloud to grow and the growth is a function of the time step; the large size of the final tracer cloud for the 5 day model, compared to the longer *dt* models, attests to this.

Across‐streamline and along‐streamline dispersion is analyzed because the streamline plays a key role in the tracer transport and thus the dispersion. In observational drifter studies, dispersion can be measured relative to potential vorticity contours in the meridional and zonal directions (Klocker et al., 2012; LaCasce, 2000). We determine the perpendicular and parallel dispersion components relative to the tracer's center of mass using:

(5) $$D_{⊥}=\frac{\sum {\left(c_{i}x_{⊥}\right)}^{2}}{\sum c_{i}}$$

(6) $$D_{∥}=\frac{\sum {\left(c_{i}x_{∥}\right)}^{2}}{\sum c_{i}}$$

where *c_i_* are the tracer concentrations in each cell and *x* are distances from the center of mass; $x_{∥}$ is the along‐streamline distance between the cell center and tracer center of mass while $x_{⊥}$ is the distance between the cell center and the nearest point on the streamline.

For all parameter choices, the dispersion is larger in the parallel than the perpendicular direction by an order of magnitude (Figures 5b–5g). The cloud size is skewed toward the higher stream function contours in the center of the gyre. In the flow used here flow speed increases in the direction in which the cloud is traveling (toward the western boundary current). Perpendicular leakage places the tracer in the inner, faster regions, where it will be transported along the flow at a higher speed. Likewise, dispersion in the outer direction will transport the tracer into a slower regime, causing a lag in the tail of the tracer cloud. Hence, the artificial dispersion is a function of the flow field also, which defines the skewness of the cloud

Next, the effect of *dx* is considered (keeping *dt* constant at 60 days; Figures 5f and 5g). As cell size increases, the amount of artificial dispersion increases. As before, the cloud grows at each time step and tracer “leaks” into adjacent cells. However, the size of each cell will affect the magnitude of dispersion; using larger resolution further increases the area covered by tracer and thus increases the dispersion. While using shorter time steps allows more spurious connections between adjacent cells to be included in the transition matrix, using larger cell sizes exaggerates the area covered by tracer as a result of these connections.

An analytically correct output would constrain the output to one streamline‐following cell. Here the model has deviated both parallel and perpendicular to the analytical solution. Moreover, an analytically correct output would lead to the same solution between each time step (equation (2)). We suggest that the artificial dispersion is an underlying cause of non‐Markovian nature in any system which maps trajectories—curved or linear—onto grids. This behavior can be reduced by increasing the time step length and reducing the cell size used.

### 3.2 Plastic Accumulation Regions

As observed in the idealized Stommel flow, the accuracy of tracer predictions depends on the transition matrix parameters. Artificial dispersion leads to an increase in the number of cells containing tracer. However, it is not immediately clear if this artifical dispersion manifests itself to the same extent when studying regional ocean flow using the more convoluted observational drifter data set, and whether it is noticeable in regional and global studies of surface dispersion.

The real ocean differs from the analytical solution in two main ways. First, the analytical scenario contains 1,000s times more trajectories, and therefore more connections between cells. Some of these connections include those that lead to artificial dispersion. Second, turbulence and submesoscale effects contribute to dispersion which is both parallel and perpendicular to the time‐averaged pathway of a drifter; this makes it more difficult to differentiate between natural, convoluted motion and artificial dispersion. The turbulence's signal may dominate over model error.

**Figure 6:**
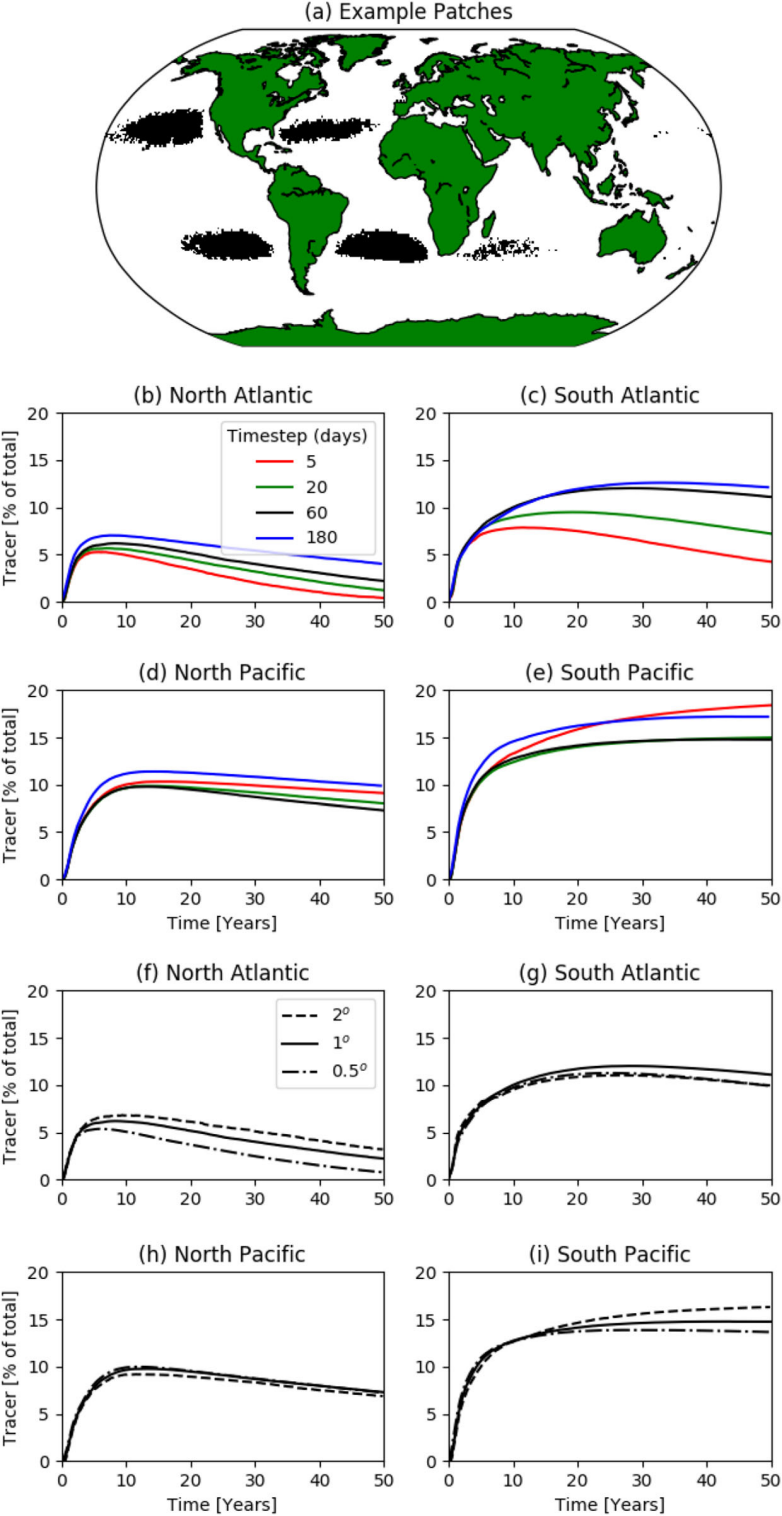
Sensitivity analysis of transition matrix parameters on ocean circulation. Tracer is initially released uniformly throughout the ocean and the system is evolved for 50 years. (a) Basin‐scale accumulation “patches”—defined as regions which have tracer concentrations larger than twice the global average. Examples are shown for the *dt* = 60 and dx=1° model. (b–e) The evolution of patch sizes in each ocean basin for a range of time steps, given a fixed cell size (1°). (f–i) The evolution of patches for a range of cell sizes, given a fixed time step (60 days). The Indian Ocean patch is omitted from the study as it does not appear in some model runs.

To test the parameter sensitivity on global surface circulation, we repeat global release experiments which were designed to detect the largest regions of surface accumulation of floating plastic litter in the ocean (Maximenko et al., 2012). These regions have been compared to observations of plastic accumulation (Law et al., 2014). Here a patch is defined as an area of ocean with tracer concentration larger than twice the mean concentration (Figure 6a; see also Van Sebille et al., 2012). At initialisation, tracer is evenly distributed throughout all ocean grid cells—hence we study the convergent nature of the entire ocean surface.

Markov models are evolved for 50 years—greater than the basin‐scale convergence time scale of ocean plastic (Van Sebille, 2014). We first compare the sizes of patches between models of different time steps (keeping cell size constant; Figures 6b–6e). The southern hemisphere gyres contain the majority of tracer; in agreement with Maximenko et al. (2012), the South Pacific retains the most tracer in this homogeneous input scenario. In Van Sebille et al. (2012), it is the second most convergent region due to a more realistic, Northern Hemisphere biased release of plastic tracer.

In all gyres, for the first 5–10 years, the accumulation is consistent between time step. Beyond this time scale, the agreement breaks down—this is independent of whether the gyre is still accumulating (South Pacific) or is losing tracer to other regions (North Atlantic).

The evolution and 50 year patch sizes vary between time steps. Given the results of section 3.1, it could be hypothesized that shorter time steps yield less accurate solutions. However, patch size is not always proportional to time step. This is made clear in the two Pacific gyres, for example, where *dt* = 180 days produces less accumulation than *dt* = 5 days but greater than *dt* = 20 days and *dt* = 60 days. Therefore, certain matrix configurations do not necessarily lead to poorer representations of ocean structures. The sparsity of plastic waste concentration measurements throughout the ocean hinders our ability to validate model output (Eriksen et al., 2014; Van Sebille et al., 2015;). It is clear, however, that there is up to a factor of 2 difference in the patch sizes, between models with different time steps, after 50 years.

Despite the results of the cell size analysis for the Stommel flow—which stated that smaller cells yield less artificial dispersion—here we capture very similar patch growth for a range of spatial resolutions (Figures 6f–6i). Thus, the increase of tracer cloud size when using larger cells does not change the model's ability to recreate surface accumulation.

Unlike for the analytical Stommel flow, it is not obvious that a particular parameter choice performs best at simulating oceanographic structures. While it is not possible to discount the presence of artificial dispersion in case studies using real drifter data, it seems its contribution is not clear. One reason may be that, during the construction of transition matrices, using longer time steps reduces the number of available subtrajectories (a reduction of 40% from 5 to 180 days); this reduces the quality of ocean current representation. So, while artificial dispersion is larger for smaller time steps, larger time step matrices contain less information on surface phenomena. Hence, the artificial dispersion and the quality of representation may counter each other. Over the time scale of 50 years, the accumulation of ocean currents perhaps dominates over the divergent nature of artificial dispersion. Therefore, moving forward, it is not possible to choose a single time step as the most representative so, throughout our further analyses, we compare results for a range of time steps.

## 4 Interocean Exchange Case Studies

A key theme in transition matrix representations of the global ocean has been the connectivity between ocean basins. Maximenko et al. (2012) define several ocean basin based on minimal interbasin transport time; Froyland et al. (2014) decompose a transition matrix into its eigenvectors, which represent the most convergent nodes in the ocean network (i.e., the basin accumulation centers); Ser‐Giacomi et al. (2015) apply an extensive range of network theory techniques to the Mediterranean Sea. Here we also focus on exchanges of surface flow at key regions of interocean connectivity, by exploiting the efficiency and simplicity of the Markov Chain model.

### 4.1 Agulhas Current System

Flow from the Agulhas Current splits into two pathways near the retroflection: (1) Agulhas leakage into the South Atlantic through Agulhas rings and other coherent structures and (2) the Agulhas Return Current into the Indian Ocean. Of drifters which enter through the Agulhas Current, 25% leave through Agulhas leakage and the rest enter the Return Current (Van Sebille et al., 2009). Agulhas rings are transient features; 3–6 are formed annually when the retroflection reaches an unstable state (Boebel et al., 2003a). As they transport water northwest away from the retroflection, into an area rich in submesoscale activity, the rings decay before reaching the South Atlantic subtropical gyre (van Sebille et al., 2010). Despite the decay of coherent structures, which initially carry water and tracer into the Atlantic, the pathways of tracer continue northward; Rühs et al. (2013) find Agulhas‐to‐Gulf Stream transport time scales on the order of a decade. It is therefore crucial for ocean transport models to capture the rich dynamics of the area.

Here tracer is released at (31°E, 32°S), at *t*_0_, within the Agulhas Current, upstream of the average position of the Retroflection (Beal et al., 2011). We define as Agulhas leakage all tracer which flows east of the Good Hope line, which is used in mooring and research cruises Ansorge et al. (2005), while tracer which flows west of 40°E is taken as the Return Current.

**Figure 7:**
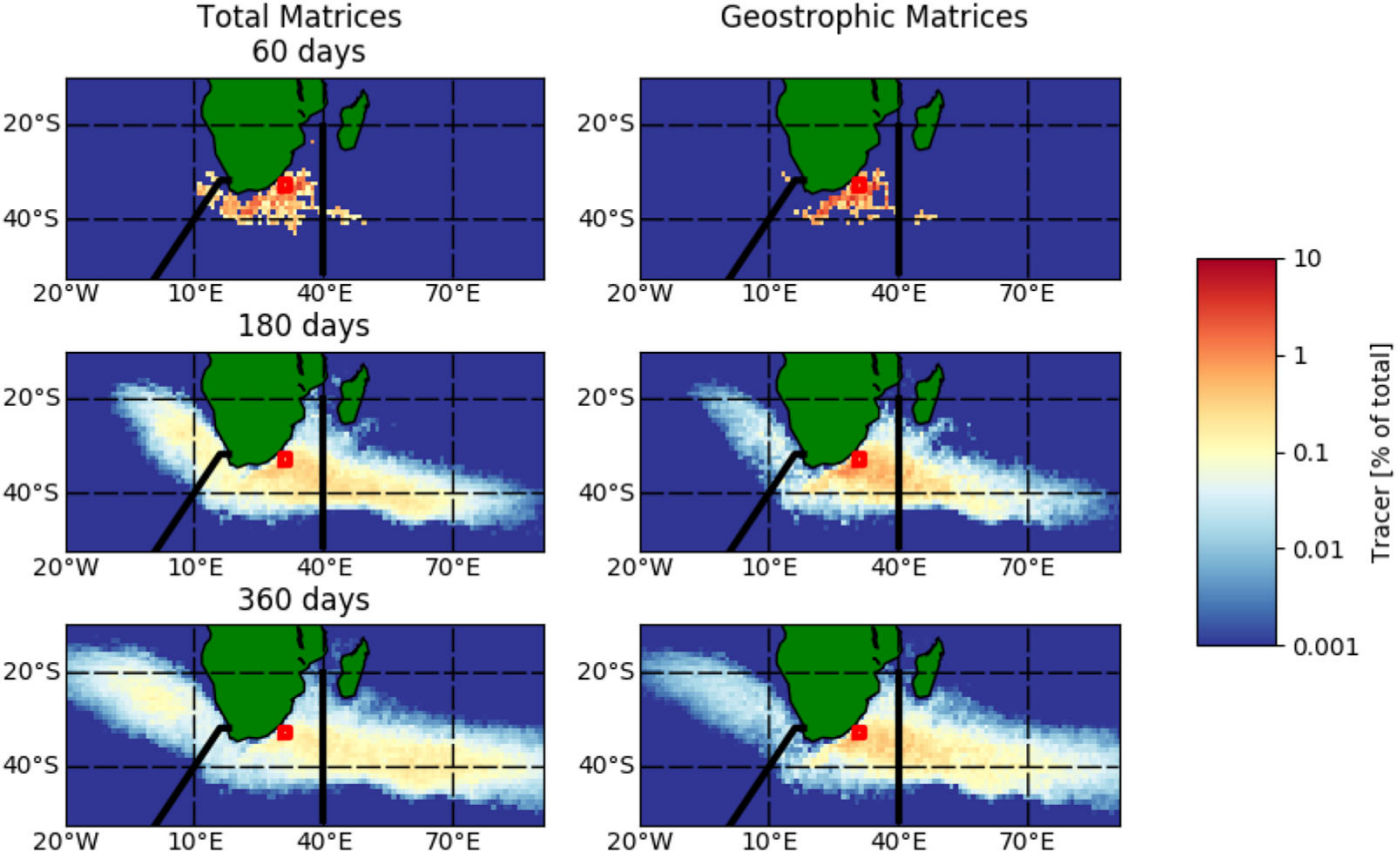
Distribution of tracer in the Agulhas Current System. Flow exits the region via the Agulhas Retroflection Current (east of 40°E) or Agulhas leakage (west of the Good Hope line). Tracer is released from the red square, within the Agulhas Current and evolved using $T_{60\;\text{days}}$.

In both the full‐field and geostrophic models, the tracer cloud grows perpendicular to the path of the Agulhas Current, as well as being advected along it (Figure 7, left column). Note that this has occurred after one time step (60 days), implying that the artificial dispersion is not the reason behind the growth of the tracer cloud but rather dispersion by eddies. After 60 days, the tracer separates at the Retroflection but even after a year, two distinct clouds have not been formed.

The Return Current, observed to be 60–80 km wide, meanders up to 3° before 40°E (Boebel et al., 2003b). However, between the source and the Return Current at 40°E, the drifter tracks display a recirculation in agreement with (Van Sebille et al., 2009)—allowing tracer to move to and from the source and Return Current area. Additionally, mesoscale eddies formed by the meandering of the Return Current may return drifters to the source regions. This explains how tracer is maintained in the source region (Figure 7; Boebel et al., 2003b). For the Agulhas leakage, though the individual rings and filaments are narrow (∼50 km) features, they do not disperse in a single direction but instead spread (and decay) throughout the South Atlantic. Hence, the time‐averaged flow field of the leakage will represent a broad cloud of tracer.

**Figure 8:**
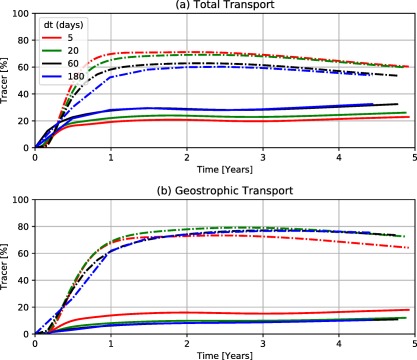
Transport of tracer in the Agulhas Current System, past the Agulhas leakage (bold line) and Return current (dashed) boundaries as defined in Figure 6. (a) Surface transport using full‐field Markov Chain models with different time steps. (b) Same as Figure 8a, but for geostrophic Markov Chain models. Van Sebille et al. (2009) found 25% of surface drifters leave via Agulhas leakage, with 75% leaving via the retroflection.

Fluxes are in agreement with the observations: 18–25% is leaked into the North Atlantic compared to 25% of the drifters in (Van Sebille et al., 2009), while 55–61% enters the retroflection (Figure 8a). The remainder recirculates within the Agulhas region. The quantity of tracer, in each area, plateaus after 1 year—representative the time taken for the initial tracer to exit the region. However, some tracer reenters the region; the tracer quantity in the Return Current begins decreasing by ∼10% after 2.5 years, which is explained by the recirculation to the Agulhas region.

The comparison of a geostrophic field with the full‐flow field can highlight which features in the transport regime are dominated by geostrophy. The Agulhas leakage serves as an example; the size of the time‐average tracer cloud and the tracer proportions within it are lower when nongeostrophic transport is omitted. Hence, while the formation of Agulhas leakage structures is caused by the instability and break‐off of a geostrophic current (the Agulhas Current), the features themselves undergo large changes in ADT during their decay. The tracer distribution shifts from (approximately) 25% to 15% in the leakage, and 70% in the Return Current. The exception is for the $T_{dt=5\;\text{days}}$ model, which is more similar to the full‐flow field results; this may be due to either the misidentification of geostrophic trajectories (see final paragraph of section 2.3) or the artificial dispersion, both of which are more prominent when using a shorter time step.

To assess whether the size of the drifter data set is adequate in depicting fluxes, we repeat the Agulhas Current experiment for transition matrices derived from two virtual particle data sets (supporting information). The total surface velocity field, geostrophic plus nongeostrophic, taken from the 0.25° GlobCurrent database (Rio et al., 2014), was implemented into the Parcels tracking tool (Lange & van Sebille, 2017). The first data set has a similar number of drifters to the GDP data set, and the second has an order of magnitude more. Transition matrices are created with both data sets; the resulting Markov Chain model fluxes into the Agulhas leakage and Return Current do not differ, between the data set‐derived models, by more than a percent (supporting information Figure S5). The near‐identical results suggest that the coverage of the GDP data set is suitable for constructing Markov Chain models which capture enough information on the surface transport. The sensitivity of the fluxes to the choice of time step is still evident when using virtual trajectories. Differences between GlobCurrent‐derived fluxes and the GDP‐derived fluxes may be due the GlobCurent products' inability to represent submesoscale phenomena.

### 4.2 North Atlantic Transport Barrier

The Gulf Stream Extension and North Atlantic Current are the northern boundary of the North Atlantic subtropical gyre and form a barrier for transport between this and the subpolar gyre. Between the North Atlantic subtropical and subpolar gyres, only one surface drifter has traversed the Gulf Stream front in three decades of observations (Brambilla & Talley, 2006). The transport barrier is apparent only at the surface; model studies show that virtual drifters deployed in the subtropical regions are more likely to cross into the subpolar regions at depths of ∼ 700 m (Burkholder & Lozier, 2011; Foukal & Lozier, 2016), or, more generally, along isopycnals (Bower & Rossby, 1989). Near‐surface heat transport is a small (10%) but important proportion of the net transport; within this proportion, the mesoscale geostrophic eddy contribution is believed to be negligible (Johns et al., 2011). Therefore, ageostrophic processes may play a key role in transporting heat between the subtropical and subpolar gyres along the surface.

**Figure 9:**
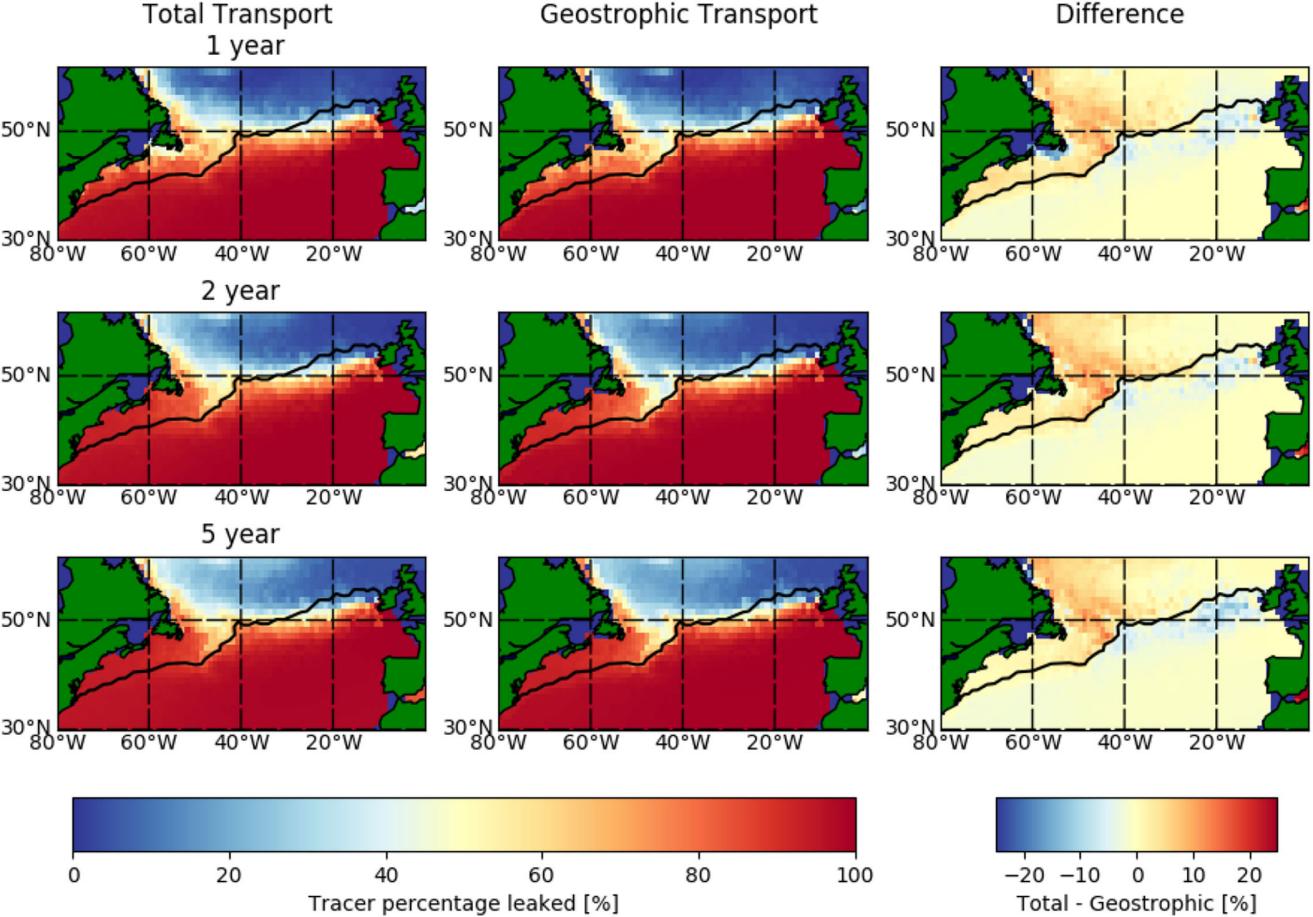
Cross‐frontal leakage between subtropical and subpolar gyres in the North Atlantic for full‐field and geostrophic drifter flows. For each cell, the quantity which remains (or enters, if beginning north of the front) in the subtropical gyre is counted. The front (transport barrier) is defined by Rypina et al. (2011) as the zero value for stream function in a drifter‐derived velocity field.

Defining oceanographic fronts is not trivial. Rypina et al. (2011) performed a drifter‐based study of the velocity field in this region and identified a stagnation (zero value) streamline which separated the contrasting subtropical and subpolar flow regimes (Figure 9, black line). Typically, fluxes into the North Atlantic are calculated across a particular latitudinal line, as opposed to a more rigorous definition of a transport barrier (e.g., Brambilla & Talley, 2006; Rühs et al., 2013). We extend the analysis of North Atlantic cross‐frontal transport to tracer which originates on both sides of the Gulf Stream, using both 45°N and the Rypina et al. (2011) stagnation streamline. Therefore, we focus on transport in both directions whereas previous studies focused only on tropical‐to‐polar transport (Brambilla & Talley, 2006; Hakkinen & Rhines, 2009). In the global drifter data set, the amount of drifters which cross the fronts on time scales from 5 to 180 days are roughly equal, with the minimum being approximately 5,000 for a 5 day period. Hence, the cross‐frontal transport in both directions is sampled evenly and adequately by the global data set.

Tracer is released in each cell in a rectangular domain extending from (80°W, 30°N) to (0°E, 60°N), individually. The one drifter which made the journey from the Gulf Stream to the subpolar gyre took 495 days to do so (Brambilla & Talley, 2006); we evolve the system beyond this time, to 5 years, to capture the change in connectivity with time. For each release experiment, we determine the quantity of tracer which traverses the transport barrier from the north, or remains south, of the transport barriers. The proportion of tracer crossing or remaining in the barriers is calculated for each cell and the experiments are repeated for both the full and geostrophic matrices and for both definitions of a North Atlantic transport boundary, using $T_{dt=60\;\text{days}}$ (Figure 9). As seen in the Agulhas Current System case study, the shorter time step model (i.e., *dt* = 5 days) approximates the full‐field model results, while the longer time step models all highlight differences between geostrophic and full‐field models (supporting information Figure S6).

For the full matrix experiments, nearly all (≥95%) of the tracer which originates south of the stagnation line remains south, in the subtropical gyre system. The western part of this barrier (before 50°W) corresponds to the Gulf Stream. Here tracer which originates off the coast of the Northern part of the U.S/Canada traverses the apparent transport barrier into the southern gyre system. Tracer which originates in the Labrador Current also reaches and remains south of the barrier. In the central North Atlantic (around 45°N, 45°W), the similarity between the stagnation line and the barrier implied by the transition matrix analysis is evident. To the east, the model‐derived barrier is further south than the stagnation line found by Rypina et al. (2011). The subpolar region is therefore partially connected to the subtropical region through the Labrador Current and this connectivity remains coherent even after a 5 year period (Figure 9, left column). The gradient from no northern tracer to no southern tracer—which runs along the Labrador Current and North Atlantic Current—is several 1° cells wide. There is no clear change in the size of this border region as the tracer is advected beyond 1 year.

In the geostrophic model (middle column in Figure 9), the tracer which originates in the tropics remains there; the tropical‐to‐polar transport blockage remains unchanged. Moreover, the area of minimal cross‐frontal leakage follows the stagnation line to the same extent as the full model. Nevertheless, there are important differences between the full‐field and geostrophic field connectivity (right column in Figure 9). There are regions where polar‐to‐tropical transport does not take place in the geostrophic model. The tracer carried by the Labrador Current is exposed to the cross‐frontal processes which occur; further east, either there is minimal transport from the poles to the transport boundary or the cross‐frontal processes are weaker. There is also equatorward transport from the coastal waters of North America, which lies north of the Gulf Stream; this is also shown for the geostrophic flow. A pathway, which exists in the geostrophic flow field, from north of the Gulf Stream to the south may involve geostrophic eddies. While the Gulf Stream is associated with relatively intense eddy kinetic energy field, this is less intense to the north of the Gulf Stream (Reverdin et al., 2003).

To confirm the presence of the transport barrier suggested by the drifter‐based transition matrix model, and the disagreement with the stagnation line, the same experiment is repeated to assess the quantity of tracer which enters/remains south of 45°N (supporting information Figure S7). The distance between the stagnation line and 45°N border is up to 15° latitude, with the former flanking the Gulf Stream and the latter cutting through it. However, the model‐derived connectivity when comparing flow past the Gulf Stream and 45°N are similar.

A caveat in this analysis is that some cross‐frontal transport will be caused by artificial dispersion, much like in the Panama case study (Figure 1). Moreover, oceanographic fronts are typically less abrupt than the geographic example, due to their motion. The North Atlantic/Gulf Stream front oscillates around the fixed boundaries (the streamline in Figure 9, or the latitudinal line in supporting information Figure S7), but the seasonal range of latitudes remains below 1° (Kelly et al., 1999), the cell size used in the Markov Chain model. In a time‐averaged transport field, this motion of the front leads to increased artificial connectivity between the regions it oscillates between. If artificial dispersion was a key component in the cross‐frontal transport there would be equal amounts of transport in both directions. However, there is less tropical‐to‐polar transport along the front. The asymmetrical connectivity suggests that there is real equatorward transport. The quantity of polar‐to‐tropical transport, as calculated by transition matrices, is nonetheless an upper estimate.

## 5 Conclusions and Discussion

This study explores the error (artificial dispersion) inherent to Markov Chain modeling and considers how this manifests itself in oceanographic case studies. First, the dispersion of a tracer cloud in an analytical, purely advective gyre system was compared for a range of transition matrix parameters (cell size, *dx*, and time step, *dt*). Artificial dispersion occurs at each model time step and is therefore more noticeable when using small time steps, while it also increases with cell size. Next, the global surface circulation pattern was recreated using observed drifter trajectories. When using the observed ocean drifters, there is no clear correlation between the parameters and the model quality. Nonetheless, the transition matrix parameters, as well as the quantity of available trajectories, affect the models accuracy in reproducing surface circulation dynamics. In light of this, we used a range of time steps in our analyses of interocean exchange.

Furthermore, this study assesses the quality of Markov modeling in depicting transport for two key regions for interocean exchange—the North Atlantic and the Agulhas Current System. In both regions, we study the role played by interocean barriers (i.e., Gulf Stream), and connections (i.e., Agulhas leakage)—both geostrophic features. By mapping altimetry‐derived values of absolute dynamic topography (ADT) to drifter coordinates, we extract a subset of drifters which remain within a narrow ADT range and thus create approximate ADT‐following transition matrices. The comparison of time‐averaged separation of the Agulhas Current in both the full and geostrophic models displays the dominance of geostrophic flow in forming the Return Current transport, and a lesser role for geostrophy in contributing to Agulhas leakage. However, the differences between full‐field and geostrophic flow models are not visible when using the shortest time step; the misidentification of geostrophic subtrajectories (equation (3)) reduces the ability of short time step models to capture geostrophic flow. Moreover, AVISO ADT fields do not resolve the smallest mesoscale, and submesoscale eddies.

Benefiting from rapid and repetitive experiments, we seed the entire North Atlantic domain with tracer to study the surface flow between the subtropical and subpolar gyres, in both directions. There is negligible poleward transport of tracer which originates in the subtropical gyre, as expected from previous understanding of the region. However, we detect surface flow from the subpolar into the subtropical gyre via the Labrador Current and to the north of the Gulf Stream, regions which were considered to be outside the subtropical gyre system. This transport corresponds to changes in ADT values, highlighting the ability of nongeostrophic processes to traverse fronts.

In this study, the sensitivity of Markov Chain model output to the transition matrix construction parameters has been quantified; the sizes of basin‐scale accumulation regions differ by up to a factor of two when comparing models with different time steps.

Considering the practicality of Markov modeling, short time steps may have more worth than longer time steps. Half‐yearly output is suitable for decadal to millennial scale studies, while regional and yearly scenarios (i.e., transfer of floating pollution between two neighboring countries) are preferably studied and visualised at finer temporal resolution. Fortunately, the apparent lack of artificial dispersion in the ocean means it seems acceptable to use weekly time steps. Due to the complex nature of ocean trajectories, it is difficult to discern between artificial dispersion and turbulence.

Surface circulation model outputs from the wide range of time steps used produce similar output over a certain time. For the global circulation, this time scale is ≈10 years (Figures 6b–6i). For the Agulhas Current System case study, it is less than 1 year (Figure 8). In the context of this paper, the concept of a Markovian time scale for the ocean would suggest the time scale at which artificial dispersion is negligible. Generally, such a quantity could indicate when and where to use this modeling technique to best effect. Crucially, the real ocean appears to be much more “Markovian” than the idealized Stommel case study.

To avoid the artificial diffusion which may arise when mapping trajectories onto a rectangular grid, a coordinate system whose cells followed the streamlines may reduce artificial dispersion (particularly in the perpendicular direction). While this is achievable for smooth, analytically derived trajectories (i.e., the Stommel intensification), such a solution is perhaps harder to visualise for the convoluted and complex trajectories found in the real ocean. However, drifters have a tendency to maintain similar ADT values for weekly‐to‐bimonthly time scales. Thus, replacing the latitude‐longitude grid with an ADT‐following grid may reduce artificial dispersion. However, the regions where the majority of drifters do not follow ADT are also key areas of interocean transport (e.g., boundary currents). Hence, in these regions, we expect there would be no improvement—in tracking transport—when streamline‐following grids are used.

The computationally cheap transition matrix method can be used to perform repeat experiments on ocean connectivity throughout basin‐sale regions, by creating connectivity maps. Brambilla and Talley (2006) and Hakkinen and Rhines (2009) both highlight the negligible surface drift from the subtropical‐to‐subpolar regions for drifters which begin in and around the Florida current; extending this analysis, we confirm the lack of poleward surface transport throughout the subtropical region and find southward transport from the Labrador Current and above the Gulf Stream (west of 40°W; Figure 9 and supporting information Figure S7). It is not within the scope of the geostrophic Markov Chain model to state which nongeostrophic processes are contributing to cross‐frontal transport. However, it has highlighted the presence of geostrophic pathways across the Gulf Stream.

## Supporting information

Supporting Information S1
