# Supplementary material for: Surface Connectivity and Interocean Exchanges From Drifter‐Based Transition Matrices
Source: J Geophys Res Oceans. 2018 Jan 19;123(1):514–32. doi: 10.1002/2017JC013363 (PMC5856081; doi:10.1002/2017JC013363)
Supplement: Supplementary file 1 — Supporting Information S1 [file JGRC-123-514-s001.pdf]

Journal of Geophysical Research Oceans

Supporting Information for

Surface Connectivity and inter-ocean exchanges from drifter-based transition matrices

Ronan McAdam[1,2], Erik van Sebille[1,2 & 3]

[1]Grantham Institute of Climate Change & Environment, Imperial College London, SW7 2AZ, UK
[2]Department of Physics, Imperial College London, SW7 2AZ, UK
[3]Institute for Marine and Atmospheric Research Utrecht, Utrecht University, 3584CC Utrecht, Netherlands

Contents of this file

Figures S1 to S7, Table S1

Introduction

The supporting information provides background on the Global Drifter Program surface drifter deployment, and further analysis of the role of transition matrix parameters in recreating ocean transport.

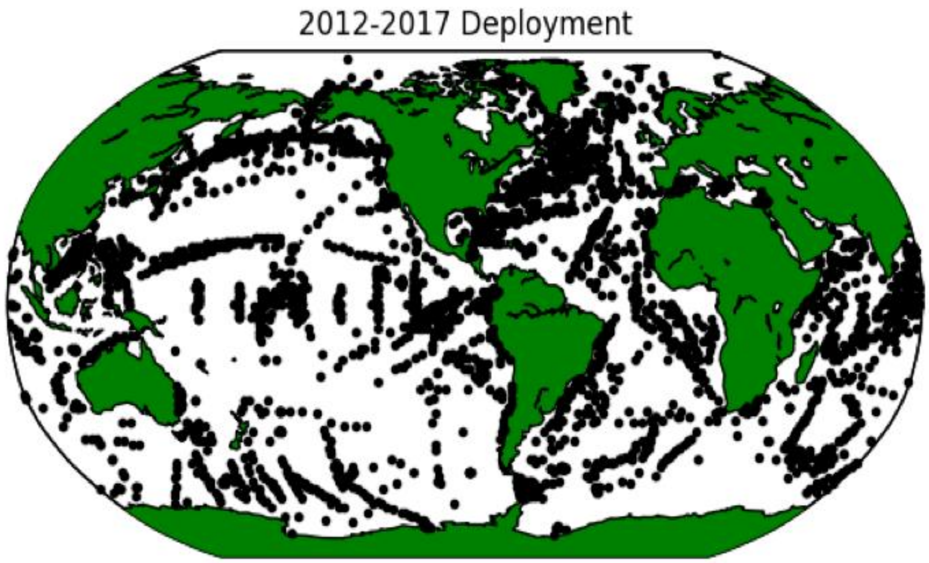

Figure S1. Global Drifter Program drifter deployment from 2012 until the most recent data release, in March 2017.

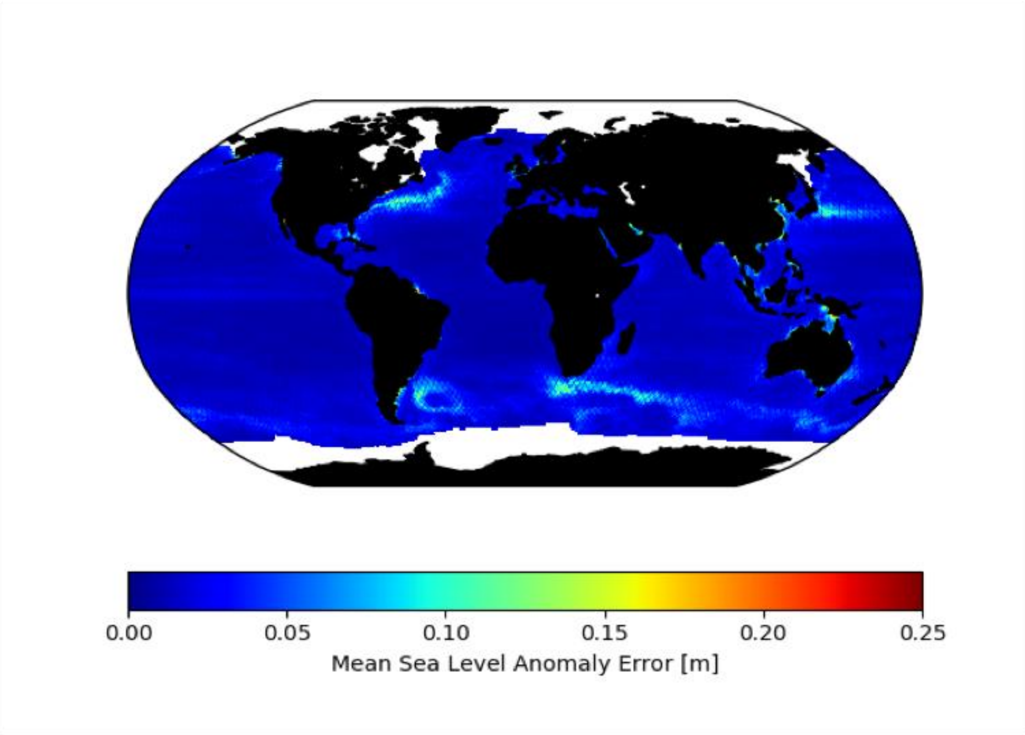

Figure S2. Mean Sea Level Anomaly (MSLA) Error, averaged from daily 0.25°-resolution maps over the 1993-2017 period, as extracted from AVISO products.

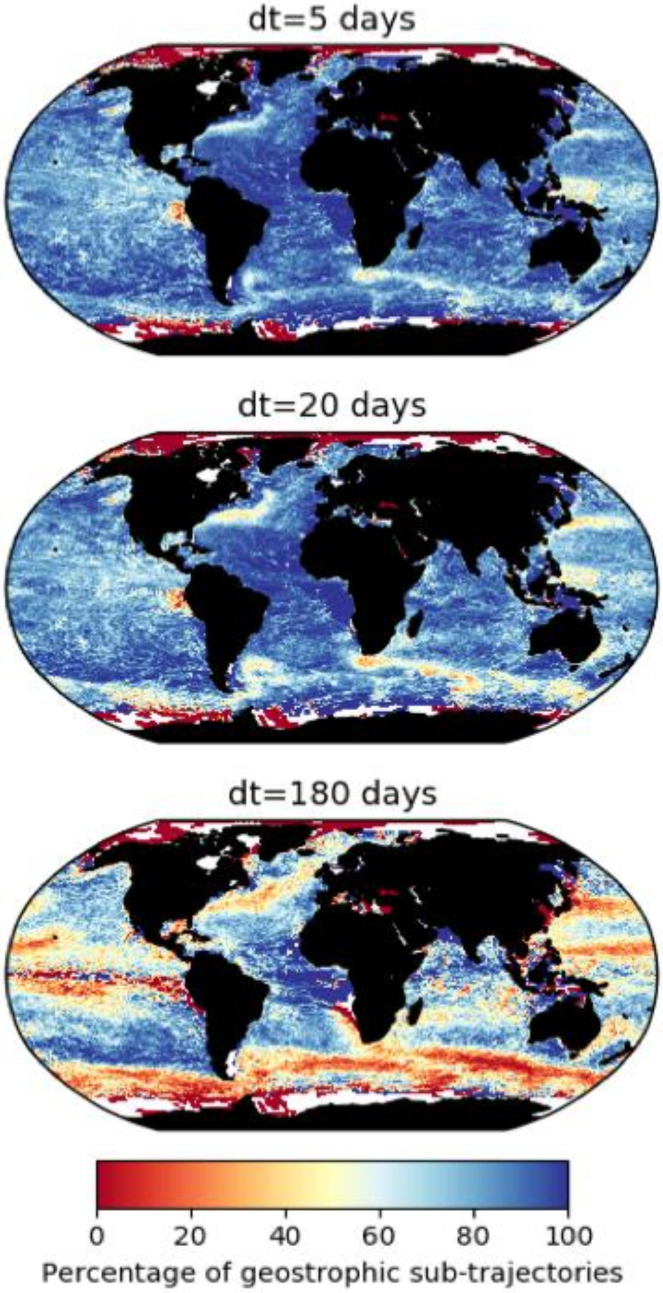

Figure S3. Maps of the proportion of sub-trajectories whose ADT values stay within 0.2m over 5, 20 & 180 day timescales (compare to Fig 3f in the main manuscript, which shows the proportion map for dt=60 day sub-trajectories.

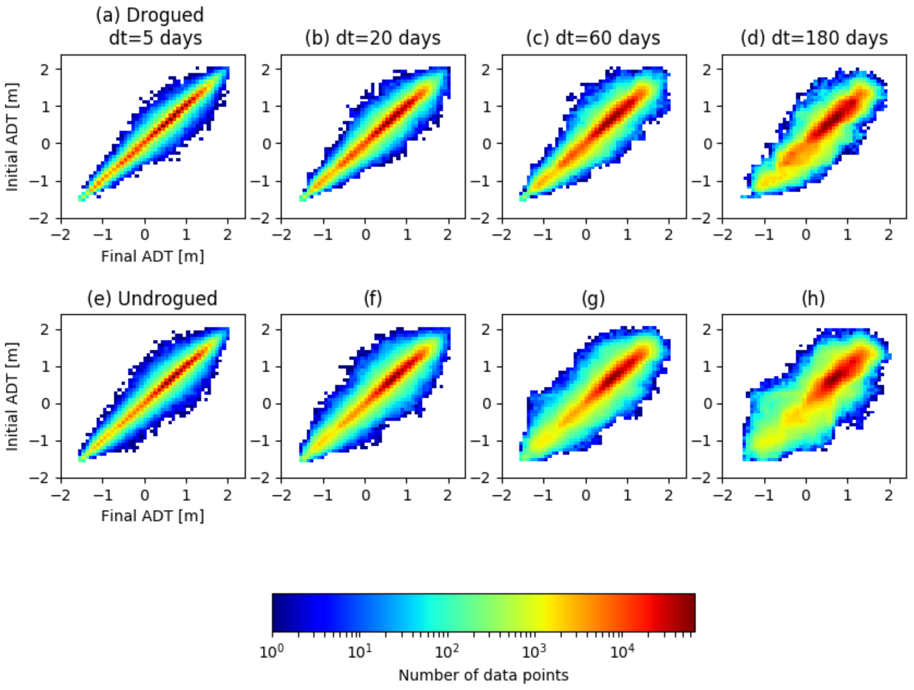

Figure S4 Mean Absolute Dynamic Topography (ADT) experienced by surface drifters. The change in ADT between the beginning and end of sub-trajectories is shown for drogued drifters (a-d) and undrogued drifters (e-h), and a range of timesteps.

%	dt = 5 days	dt = 20 days	dt = 60 days	dt = 180 days
All Drifters	*98.2*	*94.7*	*87.9*	*72.4*
Drogued Drifters	*98.6*	*95.9*	*89.7*	*76.0*
Undrogued Drifters	*97.9*	*93.8*	*86.9*	*71.6*

Table S1. Percentage of GDP drifter sub-trajectories which remain within 20cm ADT contour after a timestep *dt*.

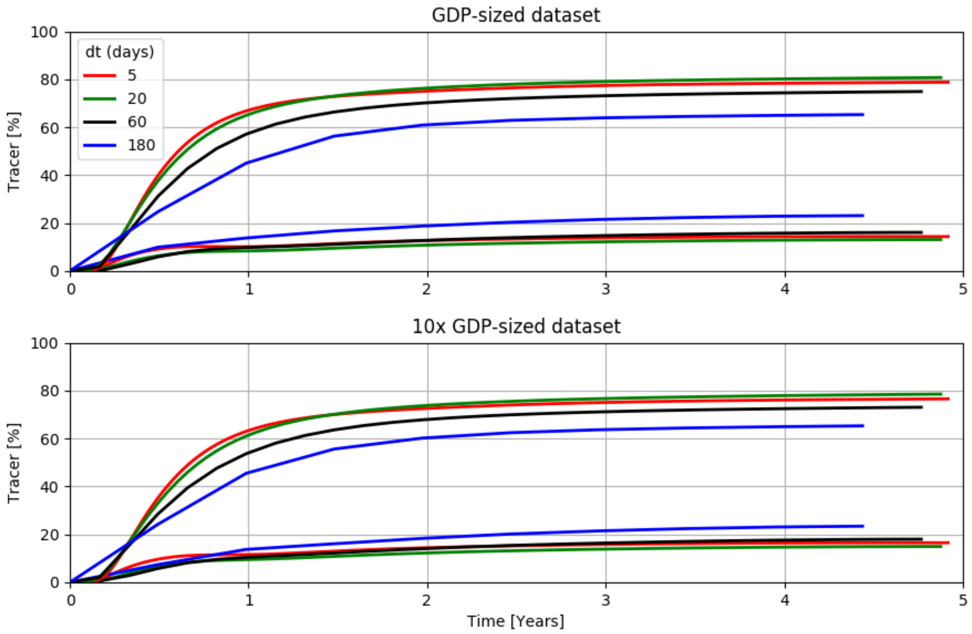

Figure S5 Transport of tracer in the Agulhas Current System, past the Agulhas Leakage (bold line) and Return Current (dashed) boundaries defined in Fig. 7. Markov Chain models are derived from two datasets which differ in the number of drifters deployed: a GDP-sized dataset (~20,000) and an order of magnitude larger (~200,000). Particles are advected along a 0.25° full velocity field at 15-m depth, from 2012 to 2014, (data produced from the www.Globcurrent.org project) using the Parcels Lagrangian toolkit (Lange, 2017). Particles are deployed on a 1.5° and 0.5° horizontal grid to provide 22,560 and 201,040 virtual drifters respectively, and advected for two years (roughly the average GDP drifter lifetime).

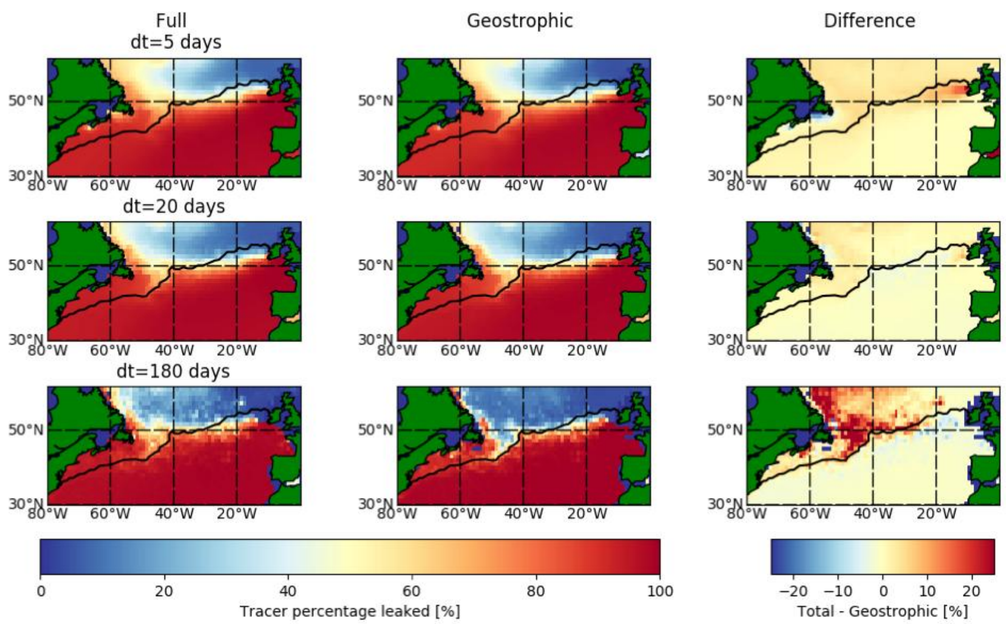

Figure S6. Cross-frontal leakage, after 5 years, between sub-tropical and sub-polar gyres in the North Atlantic for full-field and geostrophic drifters flows, using transition matrices $T_{5\ days}$, $T_{20\ days}$ & $T_{180\ days}$.

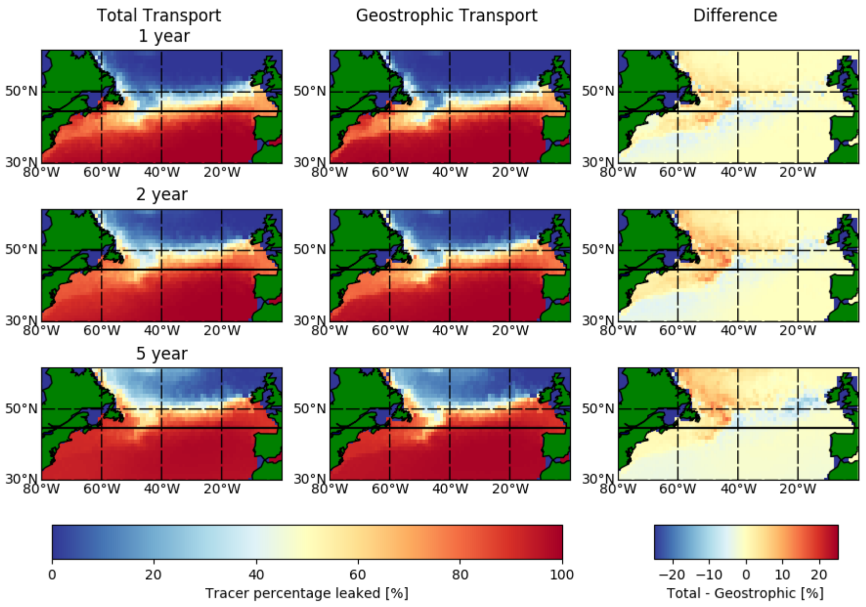

Figure S7 Cross-frontal leakage between sub-tropical and sub-polar gyres in the North Atlantic for full flow field and geostrophic flow. For each cell, the quantity which remains (or enters, if beginning north of the front) in the sub-tropical gyre is counted. The front (transport barrier) is the 45N zonal line. Compare to Figure 9 from the main article.

REFERENCES

Kelly, K. A., S. Singh, and R. X. Huang (1999), Seasonal variations of sea surface height in the Gulf Stream region, Journal of physical oceanography, 29(3), 313–327.

Lange, M., and E. van Sebille (2017), Parcels v0.9: prototyping a lagrangian ocean analysis framework for the petascale age, Geoscientific Model Development, 2017, in press, doi:10.5194/gmd-2017-167.
